# Tetrahydrofolate Attenuates Cognitive Impairment after Hemorrhagic Stroke by Promoting Hippocampal Neurogenesis via PTEN Signaling

**DOI:** 10.1523/ENEURO.0021-24.2024

**Published:** 2024-06-03

**Authors:** Xuyang Zhang, Qingzhu Zhang, Qian Zhang, Haomiao Wang, Yi Yin, Huanhuan Li, Qianying Huang, Chao Guo, Jun Zhong, Tengyuan Zhou, Yujie Chen, Zhi Chen, Qiao Shan, Rong Hu

**Affiliations:** ^1^Department of Neurosurgery, Southwest Hospital, Army Medical University (Third Military Medical University), Chongqing 400038, China; ^2^Department of Neurosurgery, The Fifth Affiliated Hospital of Zhengzhou University, Zhengzhou 450052, China; ^3^Clinical Medical Research Center, Southwest Hospital, Third Military Medical University (Army Medical University), Chongqing 400038, China

**Keywords:** cognition, hemorrhagic stroke, neurogenesis, PTEN, tetrahydrofolate

## Abstract

Intracerebral hemorrhage (ICH), the most common subtype of hemorrhagic stroke, leads to cognitive impairment and imposes significant psychological burdens on patients. Hippocampal neurogenesis has been shown to play an essential role in cognitive function. Our previous study has shown that tetrahydrofolate (THF) promotes the proliferation of neural stem cells (NSCs). However, the effect of THF on cognition after ICH and the underlying mechanisms remain unclear. Here, we demonstrated that administration of THF could restore cognition after ICH. Using Nestin-GFP mice, we further revealed that THF enhanced the proliferation of hippocampal NSCs and neurogenesis after ICH. Mechanistically, we found that THF could prevent ICH-induced elevated level of PTEN and decreased expressions of phosphorylated AKT and mTOR. Furthermore, conditional deletion of PTEN in NSCs of the hippocampus attenuated the inhibitory effect of ICH on the proliferation of NSCs and abnormal neurogenesis. Taken together, these results provide molecular insights into ICH-induced cognitive impairment and suggest translational clinical therapeutic strategy for hemorrhagic stroke.

## Significance Statement

Intracerebral hemorrhage (ICH) has been associated with cognitive dysfunction, yet its underlying mechanism remains elusive. Tetrahydrofolate (THF) has shown potential in promoting the proliferation of neural stem cells (NSCs), but its specific impact on cognitive recovery following ICH is still to be confirmed. Through the utilization of the Nestin-GFP genetic marker to track endogenous NSCs in mice, our study revealed that THF could regulate PTEN pathway to ameliorate cognitive impairment post-ICH by enhancing the proliferation of NSCs and sustaining neurogenesis. These findings contribute to valuable insights into the molecular mechanisms involved and suggest potential clinical applications for enhancing cognitive function recovery after ICH.

## Introduction

Hemorrhagic stroke is a subtype of stroke. Although the incidence of hemorrhagic stroke is only 10–20% (Hemphill et al., 2015; Mozaffarian et al., 2016), its high mortality and disability rate remain to be a clinical challenge, among which cognitive dysfunction is a major complication after intracerebral hemorrhage (ICH), the most common type of hemorrhagic stroke. Approximately 19–63.3% of patients with ICH suffer from cognitive impairment within 4 years (Planton et al., 2017; You et al., 2017; Aam et al., 2020; Kazim et al., 2021; Potter et al., 2021). With the advancements in medical care, the mortality rate after ICH has decreased, while the disability rate is still high, among which cognitive impairment is related to the decline of the quality of life of patients (Jun-Xiao et al., 2024). Despite its consequence, research efforts have paid relatively little attention to cognitive recovery after ICH. The mechanisms of cognitive dysfunction after ICH and potential therapeutic strategies are still in the early stage of exploration.

NSCs primarily reside in the subgranular zone of the hippocampus and the subventricular zone (SVZ) of the lateral ventricle (Bonaguidi et al., 2011; Bond et al., 2015). NSCs have the potential to self-renew and maintain the cell pool and differentiate into neurons and glia cells, which is called neurogenesis (Bonaguidi et al., 2011; J. Wang et al., 2019; Xuyang et al., 2024). Neurogenesis of the hippocampus is involved in the regulation of cognition, anxiety, depression, and even epilepsy (Fu et al., 2019; Terreros-Roncal et al., 2021; Zhang et al., 2021).

Phosphatase and tensin homolog (PTEN) is a classical tumor suppressor that antagonizes phosphatidylinositol 3-phosphate kinase (PI3K)/AKT signaling (Song et al., 2012). Additionally, PTEN is required to regulate the quiescence and differentiation of hippocampal NSCs and progenitors to maintain normal neurogenesis (Cao et al., 2004). Our previous findings indicate that the expression of PTEN can be regulated during the proliferation and differentiation of NSCs under oxidative stress conditions through the intervention of tetrahydrofolate (THF; Xuyang et al., 2022). THF, the active form of vitamin B9, is an essential cofactor in the one-carbon cycle and is involved in mitochondrial respiratory transport, as well as the biosynthesis of thymidylate and purine (Burgos-Barragan et al., 2017). Previous studies have shown that THF exhibits antioxidant activity (Rezk et al., 2003; Pathikkal et al., 2022), indicating its potential as a candidate for central nervous system diseases associated with reactive oxygen species (ROS) accumulation, such as hemorrhagic stroke. Therefore, we aimed to investigate whether THF could be a therapeutic candidate for post-ICH cognitive impairment through its promotion of hippocampal neurogenesis.

We hypothesized that administering THF could alleviate cognitive impairment following ICH by regulating PTEN expression. To test this, we treated ICH mice with varying concentrations of THF and assessed cognition restoration and hippocampal NSC status. Pharmacological and genetic manipulations were used to investigate the role of PTEN in cognitive dysfunction after ICH and the regulatory role of THF in this process.

## Materials and Methods

### Mouse strains and animal model

Our experiment includes the following mouse strains: C57BL/6J, Nestin-GFP, and Nestin-Cre. Adult male 2-month-old mice with a C57BL/6J background were used in our study, and all animal experiments were performed in accordance with the Chinese Protection of Scientific Research Animal Welfare Law and approved by the Animal Ethics Committee of Army Medical University (approval no. AMUWEC2020777). The mice were killed by carbon dioxide inhalation.

The basal ganglia ICH model was modeled according to our previous study (Yang et al., 2023). In brief, mice were fixed on a stereotaxic apparatus, isoflurane was anesthetized and fixed, the skin was sterilized under sterile conditions, the scalp was cut open and the skull exposed, and the location of the drilling hole was marked according to the bregma, 2 mm lateral and 0.6 mm anterior. A Hamilton microsyringe was used to extract 25 µl of blood from the tail artery and injected at a rate of 2 μl/min into the basal ganglia region at a depth of 3.0 mm. The needle was subsequently retained for 10 min and slowly withdrawn. Meanwhile, in the Sham group, only needles were inserted into the brain tissue without blood injection. After completion of the injection, the incision was sterilized and sutured, and the mouse was placed in a rewarming box for anesthesia recovery.

### EdU administration

To label NSCs proliferation in vivo, EdU (Beyotime, ST067) was dissolved in saline and injected intraperitoneally at 50 mg/kg 24 h before analysis, and to observe NSC differentiation, EdU was injected at 200 mg/kg, followed by tracing on 28 d after ICH.

### THF and afzelin administration

In vivo, THF (dissolved in saline, Sigma-Aldrich, T3125) and afzelin (dissolved in saline of DMSO, MedChemExpress, HY-N1441, 100 mg/kg) were administered 4 h after surgery by intraperitoneal injection every day for a total of 7 consecutive days.

### Immunofluorescence

Dehydrated brain tissue was sectioned coronal on a freezing microtome at a thickness of 30 μm. Brain sections were incubated with 1% Triton X-100 for half an hour at room temperature, then blocked with immunostaining blocking solution for 2 h, and stained with primary antibodies overnight at 4°C. The next day, brain sections were incubated with the corresponding immunofluorescent secondary antibodies for 2 h at room temperature. Primary antibodies are the following: rabbit anti-Sox2 (1:100, Abcam, 97959), mouse anti-GFAP (1:200, CST, 3670), rabbit anti-GFAP (1:400, CST, 12389), GFP (1:1000, Aves Labs, 1020), GFP (1:500, Invitrogen, 10262), rabbit anti-Ki67 (1:400, CST, 9129), rabbit anti-DCX (1:500, CST, 4604), rabbit anti-NeuN (1:500, CST, 24309). Secondary antibodies were applied at 1:400 dilution (goat anti-chicken Alexa Fluor 488, ab150173, donkey anti-rabbit Alexa Fluor 488, ab150173, goat anti-rabbit Alexa Fluor 555, ab150078, goat anti-mouse Alexa Fluor 555, ab150118, goat anti-rabbit Alexa Fluor 647, ab150079, goat anti-mouse Alexa Fluor 647, ab150115). Images were acquired on confocal system (LSM 880).

### Stereotactic injection of viruses

To achieve conditional deletion of PTEN in hippocampal NSCs, we injected Adeno-associated virus (AAV, virus titer 8.3 × 10^8^/ml, injection volume 300 nl) into the dentate gyrus of Nestin-Cre mice. We used a multisite injection to ensure virus infection in the dentate gyrus of the hippocampus. Our coordinates served as a reference point for the anterior 2.0 mm, lateral 1.9 mm, and depth displacement of 2.0 mm. The second coordinate point indicated a posterior displacement of 2.0 mm, lateral 1.4 mm, and depth displacement of 2.0 mm. After 21 d of infusion, mice were prepared for analysis.

### Western blot

Mice anesthetized with isoflurane were injected with saline. Then, the brain tissues were isolated and subsequently preserved in liquid nitrogen. For tissue lysis, the brain tissue was homogenized using RIPA lysate. Equal amounts of protein from each group were then separated by 10% SDS-PAGE and transferred onto PVDF (Millipore) membranes. The membranes were subsequently blocked with 10% skim milk at room temperature for a duration of 2 h. Then, they were incubated with rabbit anti-PTEN (1:1000, CST, 9559), rabbit anti-p-Akt (1:1000, CST, 4060), rabbit anti-Akt (1:1000, CST, 9272), rabbit anti-p-mTOR (1:1000, CST, 5536), rabbit anti-mTOR (1:1000, CST, 2983), mouse anti-GAPDH (1:1000, CST, 97166), mouse anti-β-actin (1:1000, CST, 3700) antibodies overnight at 4°C. Secondary anti-mouse (Boster, BA1038) and anti-rabbit (Boster, BA1039) horseradish peroxidase (HRP)-conjugated antibodies (1:10,000, Boster) were used to conjoin the primary antibodies at room temperature for 2 h. Immunoblots were detected by Image Lab software (Bio-Rad) and analyzed by Image Lab software (Bio-Rad).

### Behavioral tests

All behavioral experiments were performed 28 d after surgical manipulation. We performed the Morris water maze (MWM) test to evaluate spatial learning and reference memory of mice. MWM was performed in a round, water-filled tub (120 cm diameter). For training stage, an invisible escape platform was located 1 cm below the water surface independent of a subject's start position. Mice were placed in the water maze from different starting positions. In this way, each animal would be able to explore the location of platform, and each animal received four trials/day for consecutive 4 d. One day after the final training trial, a probe trial was performed in which the visual platform was removed.

New Object Location Task (NOL) and New Object Recognition Task (NOR): First day, mice adapted to the enclosed empty white chamber box (60 × 60 cm) freely for 15 min. After 24 h, two identical objects were placed on the one side of the empty box, and the mice were allowed to explore freely for 10 min. And then, an hour later, object recognition was tested by replacing location or a new object; the exploratory behavior was recorded with a VCR recorder. It is important to note that the new object recognition experiment was conducted 6 h after the water maze experiment, which took place 28 d following the ICH.

### Statistical analysis

All data in our work were analyzed using SPSS 23.0 and presented as mean ± SEM. The distribution of data was verified with the Shapiro–Wilk test. Independent-sample *t* tests were used for numerical comparisons between the two groups, while one-way ANOVA was used for comparisons between multiple groups and tested with Turkey's post hoc test. *p* < 0.05 was considered statistically significant.

## Results

### THF facilitates cognitive recovery after ICH

**Figure 1.**
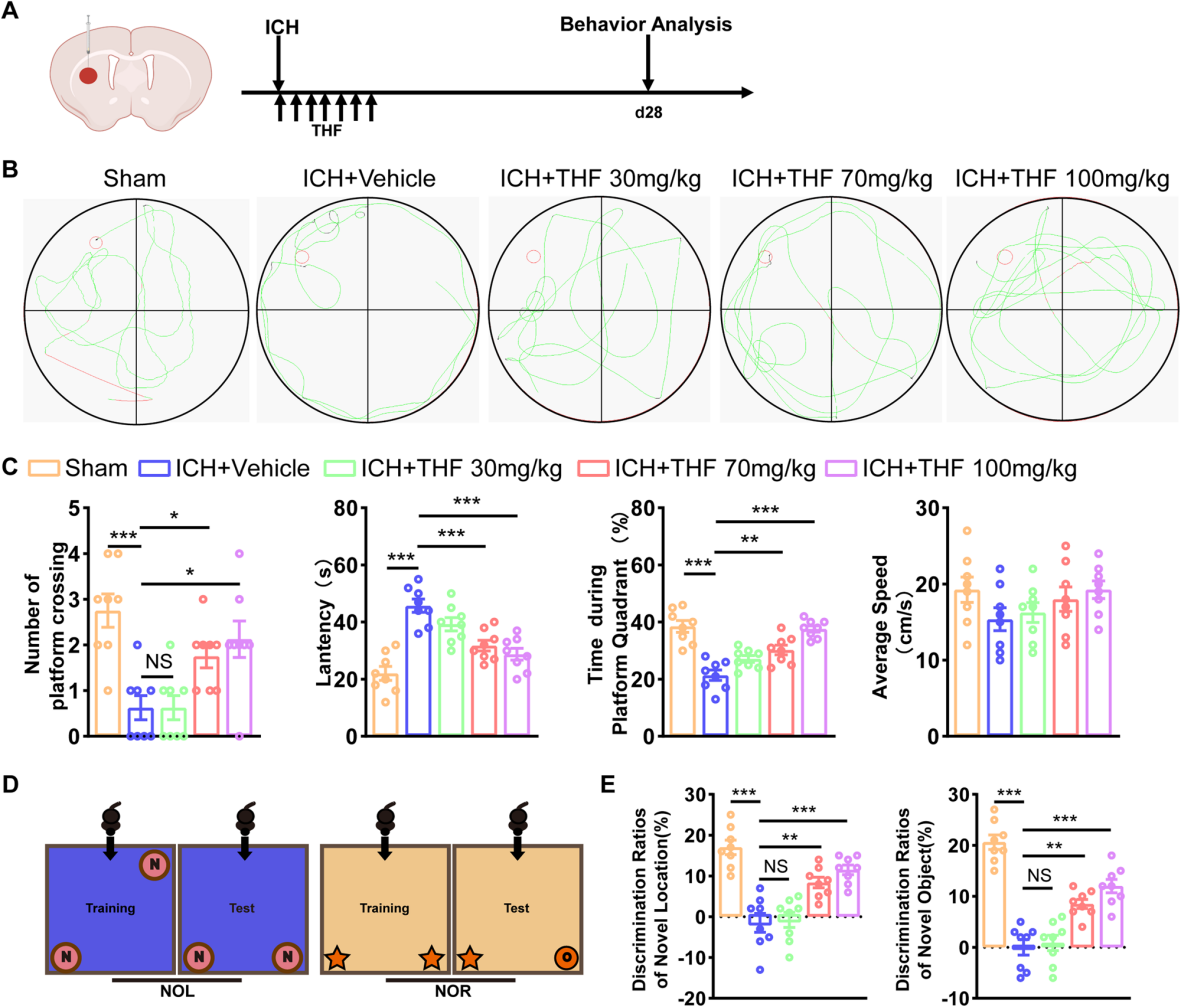
THF facilitates functional recovery in mice after ICH. ***A***, Schematic diagram of the ICH model, as well as experimental timeline for behavior test. ***B***, Representative images of the swimming path of the MWM in different groups to assess spatial learning and memory. ***C***, Platform-crossing times, escape latency, duration of stay in the platform quadrant, and average speed in different group after ICH. Mice in the ICH + THF group showed greater improvements in the MWM compared with mice in the ICH + Vehicle group. *N* = 8 for each group. ***D***, Schematic diagram of NOL and NOR test. ***E***, Quantification of the ratio of exploration time on NOL and NOR in different groups after ICH. Mice in the ICH + THF group showed greater improvements in the NOL and NOR tests compared with mice in the ICH + Vehicle group. *N *= 8 for each group. *p < 0.05; **p < 0.01; ***p < 0.001.

We first established animal ICH model using autologous blood stereotactically injected into the striatum, the main region of spontaneous deep ICH, followed by continuous THF treatment for 7 d (Fig. 1*A*). Considering the dose-dependent effect, we administered three different doses of THF (30, 70 and 100 mg/kg) to determine appropriate working concentration for post-ICH cognitive dysfunction. We assessed the effect of THF on post-ICH cognitive function using the MWM, the new object recognition task (NOR), and the new object location task (NOL), both of which are classic behavioral tests of cognitive function (Fig. 1*B*,*D*). Results from the MWM indicated that the ICH group showed significantly reduced learning and memory function compared with the Sham group, characterized by a reduction in the number of platform crossings, an increased latency to explore the platform, and a reduction in the time spent in the platform quadrant (Fig. 1*C*). Notably, the impaired cognitive function in the ICH group was partially alleviated by THF in a dose-dependent manner, with only data from treatment with 70 or 100 mg/kg THF supporting its protective role (Fig. 1*C*). Similarly, data from the NORT and NOLT showed that the ICH group had reduced exploration time of novel locations or objects and that the impairment could be partially reversed with treatment of 70 or 100 mg/kg THF (Fig. 1*E*). These findings suggest that THF has a beneficial effect on the recovery of cognitive dysfunction after ICH.

### THF promotes the proliferation of hippocampal NSCs and neurogenesis after ICH

**Figure 2.**
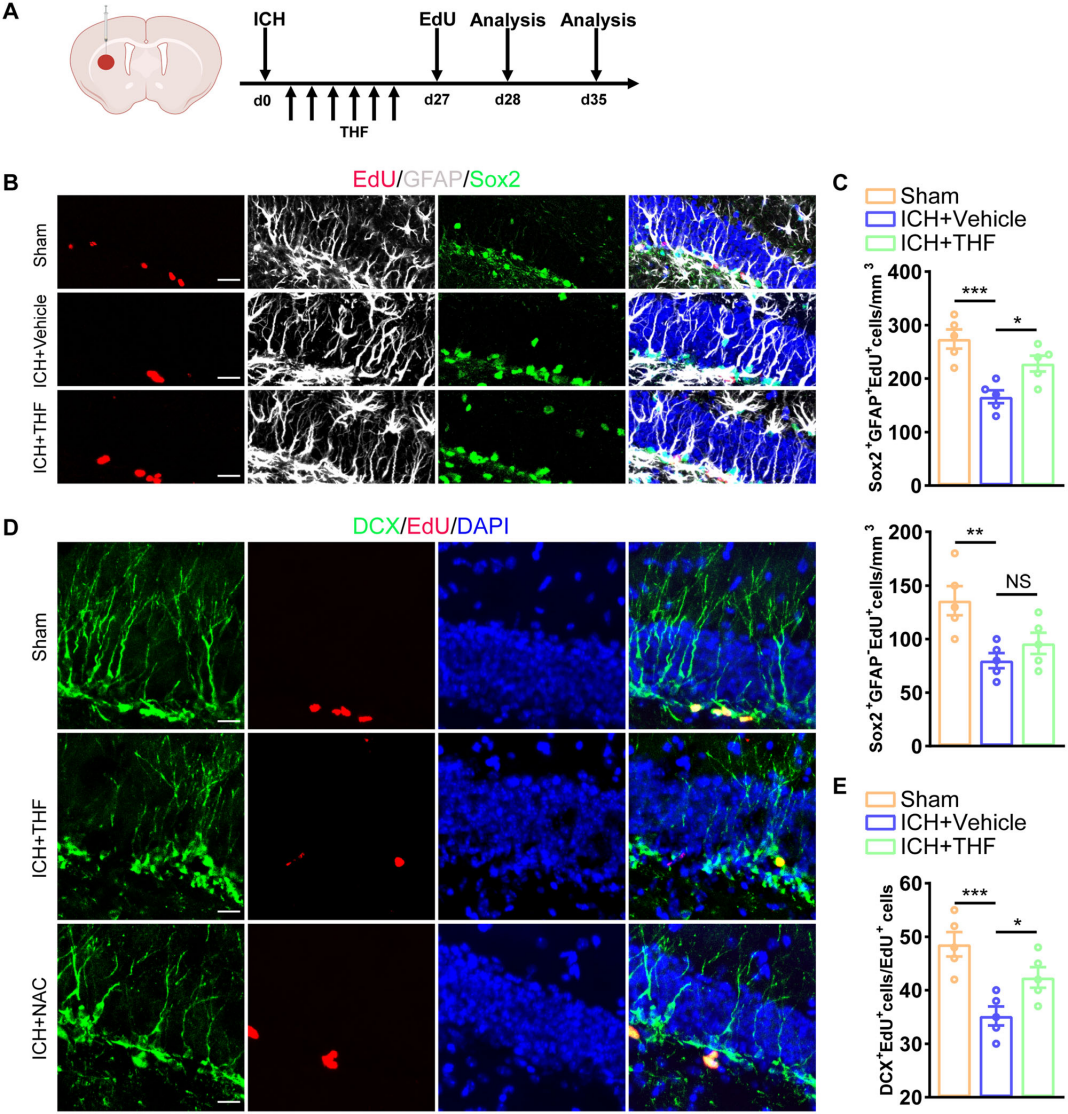
THF promotes the proliferation of hippocampal NSCs after ICH. ***A***, Schematic diagram of the ICH model, as well as experimental timeline for THF, EdU administration, and cell proliferation analysis. ***B***, Representative images of brain section stained with EdU (red, Alexa Fluor 555), Sox2 (green, Alexa Fluor 488), GFAP (white, Alexa Fluor 647), and DAPI in the DG of ICH mice. Scale bar, 20 μm. ***C***, Quantification of numbers of Sox2^+^GFAP^−^EdU^+^ and Sox2^+^GFAP^+^EdU^+^ cells from ***B***. *N *= 5 for each group. ***D***, Representative images of brain section stained with EdU (red, Alexa Fluor 555), DCX (green, Alexa Fluor 488), and DAPI in the DG of ICH mice. Scale bar, 20 μm. ***E***, Quantification of numbers of EdU^+^DCX^+^/EdU^+^ cells from ***D***. *N *= 5 for each group.

**Figure 3.**
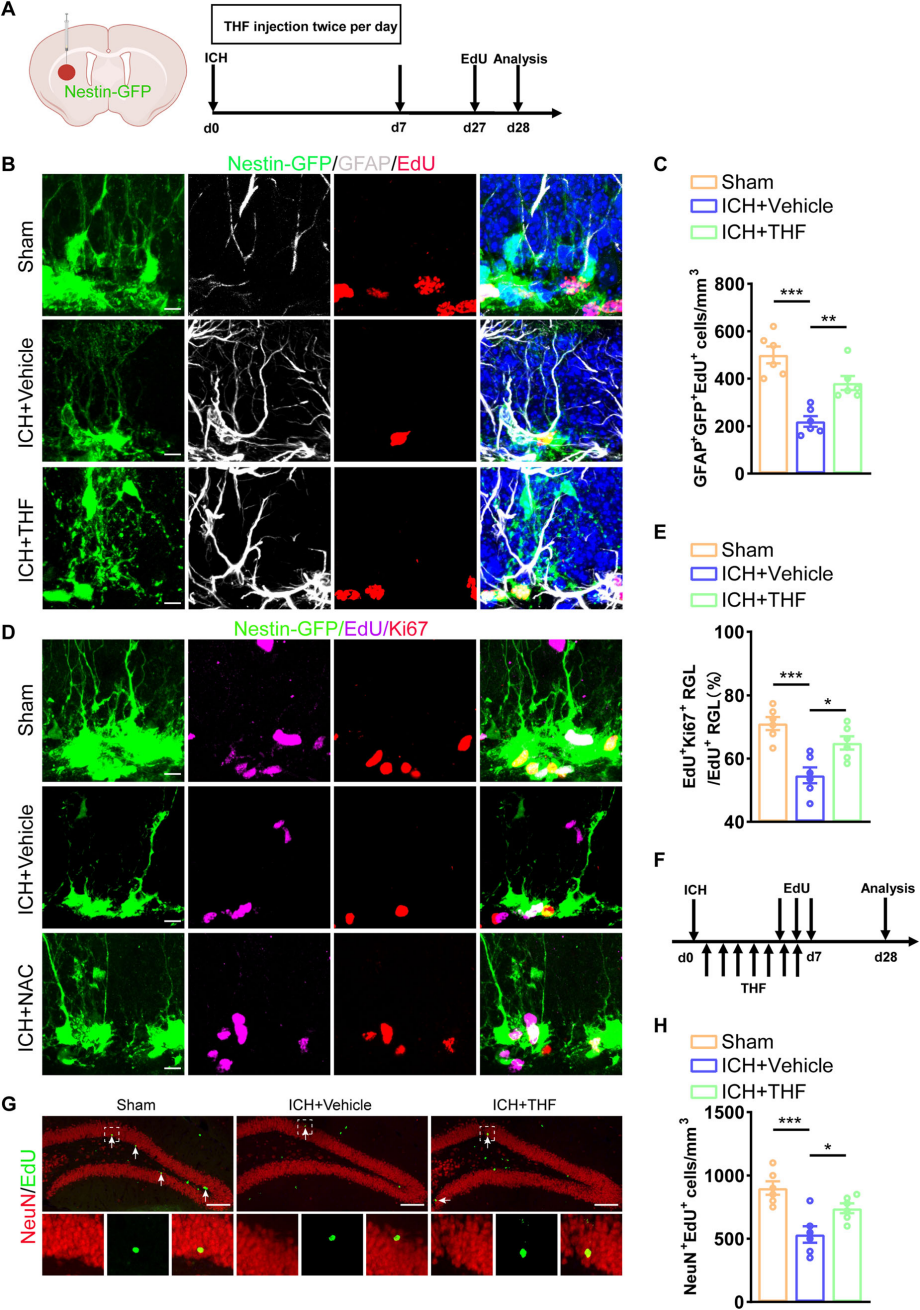
THF can alleviate ICH-induced abnormal neurogenesis. ***A***, Schematic diagram of the ICH model, as well as experimental timeline for THF, EdU administration, and neurogenesis analysis. ***B***, Representative images of brain section stained with EdU (red, Alexa Fluor 555), GFAP (white, Alexa Fluor 647), and genetically labeled GFP (green, Alexa Fluor 488) in the DG in ICH mice with or without THF treatment. Scale bar, 10 μm. ***C***, Quantification of numbers of proliferated rNSCs (GFP^+^GFAP^+^EdU^+^) cells from ***B***. *N *= 6 for each group. ***D***, Representative images of brain section stained with EdU (purple, Alexa Fluor 647), Ki67 (red, Alexa Fluor 555), and genetically labeled GFP (green, Alexa Fluor 488) in the DG in ICH mice with or without THF treatment. Scale bar, 10 μm. ***E***, Quantification of ratio of Ki67^+^EdU^+^ rNSCs/EdU^+^ rNSCs from ***D***. *N *= 6 for each group. ***F***, Experiments timeline for cell differentiation analysis of Sham, ICH + Vehicle, and ICH + THF groups. ***G***, Representative images of brain section stained with EdU (green, Alexa Fluor 488) and NeuN (red, Alexa Fluor 555) in the DG in different group. Scale bar, 100 μm. ***H***, Quantification of numbers of NeuN^+^EdU^+^ cells from ***G***. *N *= 6 for each group. *p < 0.05; **p < 0.01; ***p < 0.001.

Our previous study showed that administration of THF could promote the proliferation of NSCs in vitro (Xuyang et al., 2022). We investigated whether the protective effect of THF on cognitive decline after ICH was also mediated by promoting the proliferation of NSCs in vivo. The hippocampus is the main storage site of the NSC pool and plays a crucial role in maintaining learning and memory function by continuously generating new neurons through neurogenesis. To visualize and trace the proliferating NSCs, we used the EdU pulse method to label cells undergoing mitosis 28 d after ICH (Fig. 2*A*). We observed a significant decrease in the number of proliferating radial glia cells (EdU^+^GFAP^+^Sox2^+^) compared with the Sham group, as well as a notable reduction in the number of transiently amplifying progenitor cells (EdU^+^GFAP^−^Sox2^+^). After THF intervention, the number of proliferating radial glia-like cells was significantly restored, and a similar trend was observed in the transiently amplifying progenitor cells (Fig. 2*B*,*C*). The results were verified using Nestin-GFP mice with the same EdU pulse method to label proliferating cells (Fig. 3*A*). As expected, the number of proliferating rNSCs (EdU^+^GFAP^+^GFP^+^) decreased in the ICH group at 28 d compared with the Sham group (Fig. 3*B*,*C*). To evaluate the ratio of cells that were in proliferating status 28 d after ICH, we stained Ki67 to represent cells that entered the cell cycle. The trend in EdU^+^Ki67^+^GFP^+^ cell proportions at 28 d after ICH dramatically decreased. Likewise, THF intervention successfully mitigated the inhibitory effect of ICH on the proliferation of hippocampal NSCs (Fig. 3*D*,*E*).

Previous studies have suggested that impaired hippocampal neurogenesis has a negative impact on cognitive function (Jun et al., 2019). Therefore, it is important to determine whether the positive effect of THF on NSC proliferation could translate into enough newborn neurons generation to maintain normal hippocampal neurogenesis. Using double staining with Edu and doublecortin (DCX), we showed a notable decrease in the proportion of newborn neurons following ICH; however, the proportion of newborn neurons significantly increased after THF administration (Fig. 2*D*,*E*). Additionally, it would make more sense to EdU label adult-born neurons during THF treatment and examine the number of EdU^+^ mature neurons at Day 28, so we inject EdU during THF treatment. Finally, we detected the number of newly formed mature neurons in the ICH + Vehicle group was significantly reduced, and this effect was reversed by THF administration (Fig. 3*F*,*G*). These findings indicate that hippocampal NSCs within the niche affected by ICH exhibit reduced proliferating capacity, resulting in impaired neurogenesis and reduced generation of new neurons. Fortunately, THF appears to be an effective drug for targeting the impaired proliferation of hippocampal NSCs and restore normal neurogenesis after ICH.

### THF promotes neurogenesis by downregulating PTEN expression after ICH

**Figure 4.**
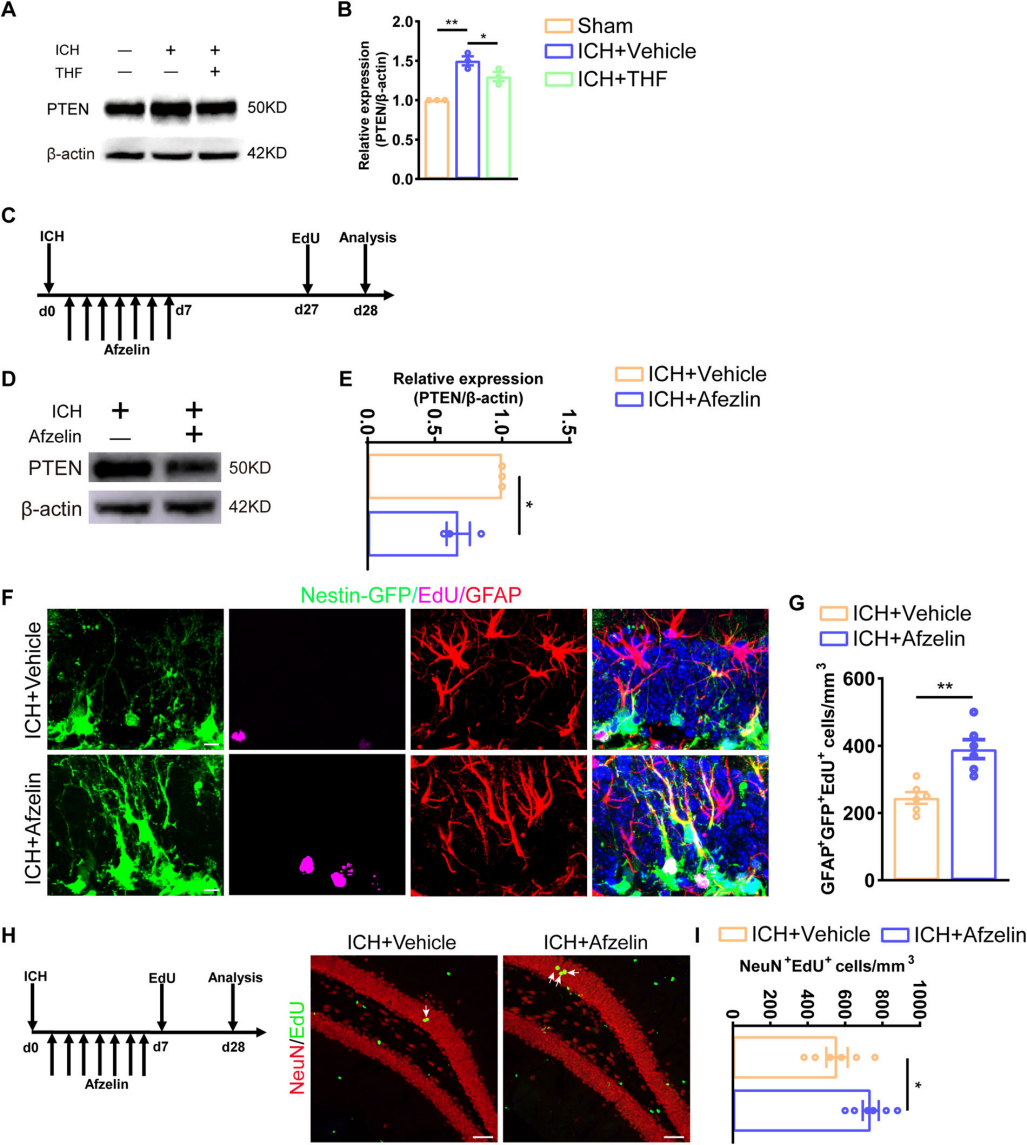
THF promotes neurogenesis by downregulating PTEN after ICH. ***A***, Immunoblot bands showing the expression level of PTEN in group Sham, ICH + Vehicle, and ICH + THF, respectively. β-Actin was served as an internal control. ***B***, Semiquantitative analysis of PTEN expression from ***A***. *N *= 3 for each group. ***C***, Schematic diagram of the ICH model, as well as experimental timeline for afzelin, EdU administration, and neurogenesis analysis. ***D***, Immunoblot bands showing the expression level of PTEN in group ICH + Vehicle and ICH + Afzelin, respectively. β-Actin was served as an internal control. ***E***, Semiquantitative analysis of PTEN expression from ***D***. *N *= 3 for each group. ***F***, Representative images of brain section stained with EdU (purple, Alexa Fluor 647), GFAP (red, Alexa Fluor 555), and genetically labeled GFP (green, Alexa Fluor 488) in the DG in ICH mice with or without afzelin treatment. Scale bar, 10 μm. ***G***, Quantification of numbers of and GFP^+^GFAP^+^EdU^+^ cells from ***F***. *N *= 6 for each group. ***H***, Representative images of brain section stained with EdU (green, Alexa Fluor 488) and NeuN (red, Alexa Fluor 555) in the DG in different group. Scale bar, 50 μm. ***I***, Quantification of numbers of NeuN^+^EdU^+^ cells from ***H***. *N *= 6 for each group. *p < 0.05; **p < 0.01; ***p < 0.001.

Previous reports have shown that THF can affect the proliferation potential of in vitro NSCs through PTEN (Xuyang et al., 2022). Therefore, we aimed to investigate whether THF could improve cognition after ICH by promoting NSC proliferation and neurogenesis via regulation of the PTEN signaling pathway. First, we used Western blotting to detect the expression of PTEN after ICH. The data showed that PTEN expression in the hippocampus was upregulated following ICH, and THF intervention counteracted this effect (Fig. 4*A*,*B*). Second, to investigate the effect of PTEN on hippocampal NSC proliferation and neurogenesis after ICH, we selected afzelin, an inhibitor of PTEN, to regulate the expression of PTEN. Afzelin is known to inhibit NOS and NADPH oxidase, which in turn decreases levels of mitophagy-related proteins including PTEN (Taslimi, 2020). Using a combination of afzelin and the EdU pulse method, we investigated the role of PTEN in regulating hippocampal neurogenesis after ICH (Fig. 4*C*). Findings from Nestin-GFP mice showed that administration of afzelin in the ICH group resulted in downregulation of PTEN expression in the hippocampus (Fig. 4*D*,*E*), accompanied by a significant increase in the number of proliferating radial glia-like cells and newly formed mature neurons (Fig. 4*F–I*). Together, these results suggest that THF might promote NSC proliferation and neurogenesis through regulation of PTEN expression after ICH.

### Conditional knockdown of PTEN promotes hippocampal neurogenesis after ICH

**Figure 5.**
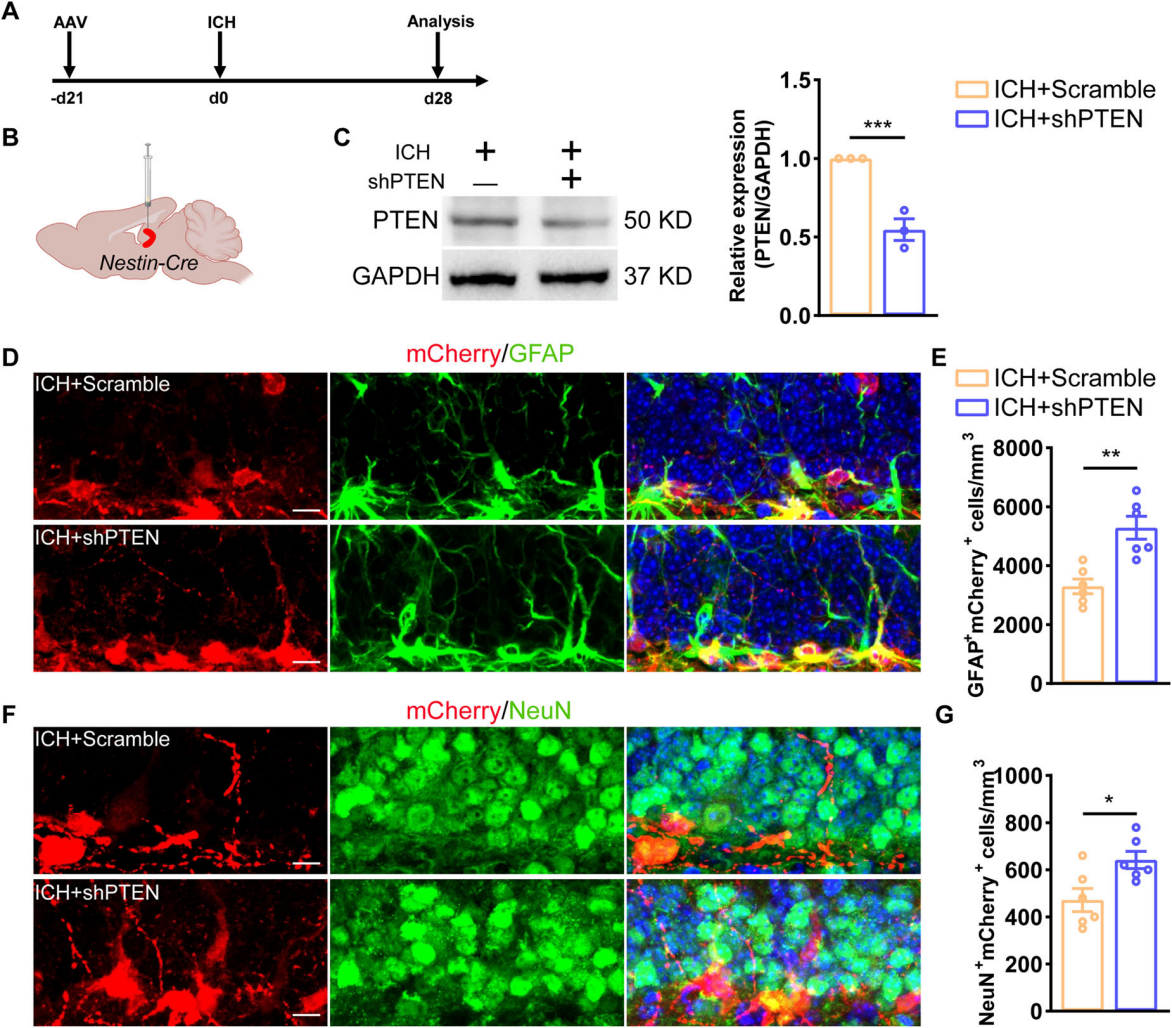
Conditional knock-out of PTEN rejuvenates neurogenesis after ICH. ***A***, ***B***, Schematic showing the stereotaxic injection of AAV into the DG of adult Nestin-Cre mice, as well as experimental timeline for analysis. ***C***, Immunoblot bands showing the expression level of PTEN in group ICH + Scramble and ICH + shPTEN, respectively. GAPDH was served as an internal control. Semiquantitative analysis of WB results. GAPDH was served as the internal control (*n*= 3 for each group). ***D***, Sample images of virus-labeled mCherry cells costained with GFAP (green, Alexa Fluor 488) in the adult DG. Scale bar, 10 μm. ***E***, Quantification of numbers of GFAP^+^mCherry^+^ cells from ***E***; *N *= 6 for each group. ***F***, Sample images of virus-labeled mCherry cells costained with NeuN (green, Alexa Fluor 488) in the adult DG. Scale bar, 10 μm. ***G***, Quantification of numbers of NeuN^+^mCherry^+^ cells from ***D***; *N *= 6 for each group. *p < 0.05; **p < 0.01; ***p < 0.001.

Next, to provide further evidence, we investigated whether abnormal neurogenesis after ICH could be reversed by manipulating PTEN expression in hippocampal NSCs. AAV that coexpresses PTEN and mCherry (CMV-FLEX-mCherry-PTEN-MCS-WPRE or CMV-FLEX-mCherry-MCS-WPRE) under lox-stop-lox boxes were injected into Nestin-Cre mice, which could produce conditional knockdown of PTEN in NSCs by binding to Lox sites to exert recombinase action (Fig. 5*A*). The viruses were injected and infected in vivo for 21 d before conducting model surgery. We observed effective knockdown of PTEN through Western blot analysis 21 d after virus injection (Fig. 5*C*). After 28 d of ICH, the animals were killed and the hippocampal neurogenesis was analyzed by immunostaining methods. The results showed that conditional knockdown of PTEN increased the number of radial glia-like cells (Fig. 5*D*,*E*) and led to more NSCs differentiating into mature neurons (Fig. 5*F*,*G*). These data suggest that selectively downregulating PTEN expression in NSCs promotes NSC proliferation and neurogenesis after ICH.

### THF upregulates downstream signaling of Akt/mTOR after ICH in vivo

**Figure 6.**
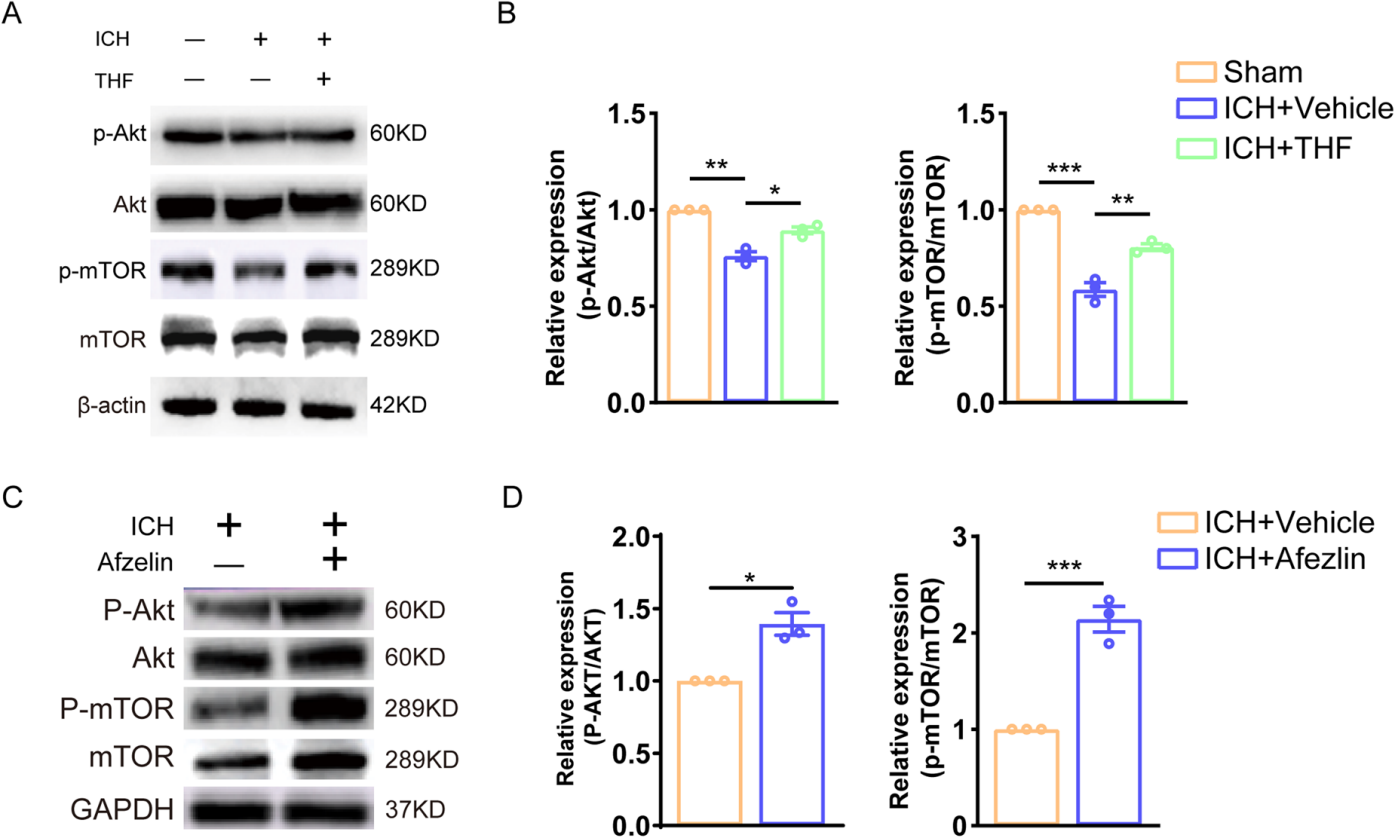
Administration of THF after ICH activated the downstream signaling pathway of PTEN. ***A***, Immunoblot bands showing the expression level of p-Akt, Akt, p-mTOR, mTOR in group Sham, ICH + Vehicle, and ICH + THF, respectively. β-Actin was served as an internal control. ***B***, Semiquantitative analysis of p-Akt, Akt, p-mTOR, and mTOR expression from ***A***. *N *= 3 for each group. ***C***, Immunoblot bands showing the expression level of p-Akt, Akt, p-mTOR, and mTOR in group ICH + Vehicle and ICH + Afzelin, respectively. GAPDH was served as an internal control. ***D***, Semiquantitative analysis of p-Akt, Akt, p-mTOR, and mTOR expression from ***C***. *N *= 3 for each group. *p < 0.05; **p < 0.01; ***p < 0.001.

**Figure 7.**
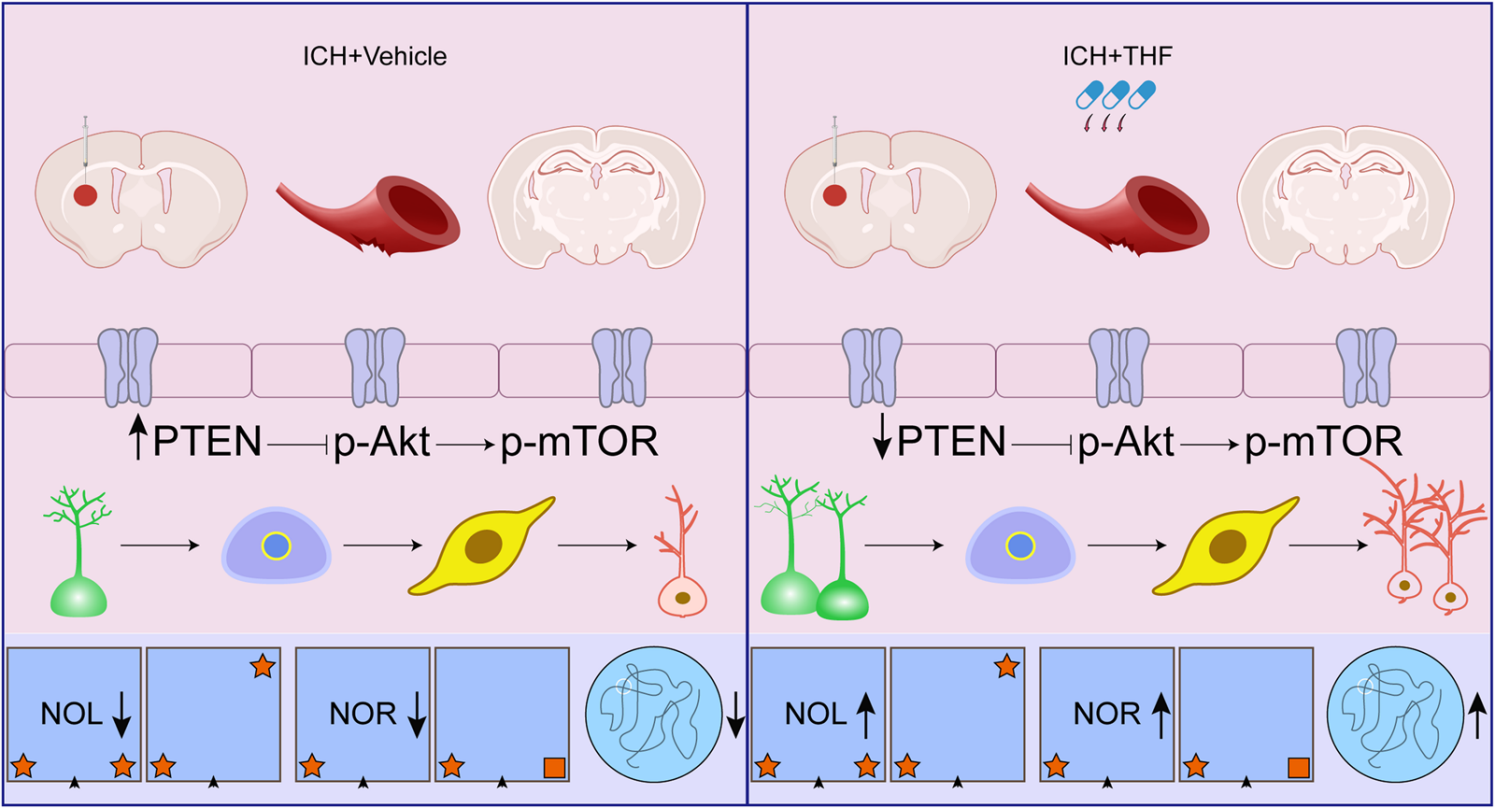
Graphical abstract illustrating the effects of administration of THF after ICH on NSCs proliferation, neurogenesis, and cognition. Administration of THF after ICH promotes NSC proliferation and neurogenesis by downregulating the expression of PTEN and upregulating the levels of p-Akt and p-mTOR, thus improving cognition.

To examine the role of downstream of PTEN signaling pathway in the effect of THF on NSC proliferation and neurogenesis after ICH in vivo, we took advantage of Western blotting assay to detect the activity of p-Akt and p-mTOR in the hippocampus following ICH. Notably, we observed an upregulation of phosphorylated Akt and phosphorylated mTOR expression in the ICH group. However, administration of THF reversed the expression of p-Akt and p-mTOR (Fig 6*A*,*B*). Furthermore, similar results were observed after PTEN inhibitor afzelin treatment (Fig. 6*C*,*D*). The results indicate that the activation of the PTEN signaling pathway after ICH inhibits neurogenesis; however, the administration of THF could restore neurogenesis and improve cognitive recovery from ICH by regulating the PTEN/Akt/mTOR signaling pathway (Fig. 7).

## Discussion

NSC therapy has shown promise in treating various neurological diseases, including neurovegetative diseases (Boese et al., 2020), traumatic brain injury (Fang et al., 2022), and stroke (Ding et al., 2023). However, the microenvironmental changes that occur after pathological damage typically impede neurogenesis and tissue repair (Ge et al., 2023), resulting in neurological deficits. Previous studies have primarily focused on illustrating the changes in NSCs located in the SVZ during ICH (Lin et al., 2023). However, these findings are insufficient in explaining the cognitive impairment following ICH. Currently, it is widely accepted that the intact construction of hippocampal neuronal circuits is responsible for maintaining learning and memory functions (Cameron and Glover, 2015). Additionally, neurogenesis is believed to contribute to the reconstruction of neuronal circuits, providing a new perspective for understanding the mechanism behind post-ICH cognitive impairment. Typically, most NSCs in the hippocampus remain quiescent (Bond et al., 2015). Upon activation, dormant NSCs enter the cell cycle and divide, renewing into new NSCs to expand the precursor population. The hippocampal NSCs also differentiate into neuroblasts and glia cells, maintaining normal neurogenesis and ensuring homeostasis in the adult brain (Bonaguidi et al., 2011; Bond et al., 2015). However, the effects of ICH on hippocampal NSCs are still unknown. Our study shows that ICH inhibits the proliferation of hippocampal NSCs and suppresses their differentiation into neurons. This may occur due to changes in the microenvironment following ICH, which trigger signals associated with cell growth and differentiation.

Numerous studies have demonstrated that folic acid supplementation can delay cognitive impairment. However, the relationship between its intermediate metabolite, THF, and cognitive function remains inconclusive. THF has been shown to reduce plasma homocysteine levels and ROS accumulation (D. Wang et al., 2016), which are considered risk factors for neurodevelopmental and neurodegenerative diseases (Jae-Won et al., 2024). This suggests a potential effect of THF on cognitive recovery. Our study found that administration of THF (70 and 100 mg/kg) significantly alleviated cognitive dysfunction induced by ICH in mice. The protective effect was mediated by promoting the proliferation of hippocampal NSCs, which effectively maintained normal neurogenesis after ICH. These results are consistent with our previous findings and support THF as a promising intervention for post-ICH cognitive impairment.

We also investigated the protective mechanism of THF against ICH-induced cognitive dysfunction, which was found to be mediated by the PTEN/Akt/mTOR signaling pathway. PTEN is a phosphatase enzyme that dephosphorylates phosphatidylinositol (3,4,5)-trisphosphate (PIP3), thereby inhibiting the PI3K/Akt signaling pathway. By doing so, PTEN negatively regulates Akt activation and downstream signaling, including the mammalian target of rapamycin (mTOR) pathway. Dysregulation or loss of PTEN function can lead to increased cell proliferation, survival, and migration, contributing to the development and progression of various diseases, including cancer (Rademacher and Eickholt, 2019; Jandy et al., 2022). Our results showed that PTEN expression was upregulated and p-AKT and p-mTOR expression was downregulated after ICH, which corresponded to the inhibition of proliferation of NSCs and impaired neurogenesis. Administration of THF reverses the neurogenesis after ICH. This effect may be attributed to the regulation of PTEN by THF, leading to the promotion of neurogenesis. PTEN plays a crucial role in the control of cell growth and differentiation, and its regulation by THF may contribute to the restoration of neurogenesis in the aftermath of ICH.

To further elucidate the underlying molecular mechanisms, we performed conditionally knock-out PTEN of NSCs of the hippocampus and found that the number of mature neuron transformation from NSCs was increased after ICH. This also suggests that PTEN plays a key role in neurogenesis after ICH. Previous studies have reported that cerebrovascular specific deletion of PTEN activates hippocampal NSCs in the early stage, disrupts neurogenesis at later stage, and ultimately results in deficits in learning and memory (J. Wang et al., 2019). Similar study has also reported that conditional PTEN deletion in RGLs initially promotes their activation and symmetric self-renewal but ultimately leads to terminal astrocytic differentiation and RGL depletion in the adult hippocampus (Bonaguidi et al., 2011). Our study demonstrates that upregulation of PTEN in the late stage of ICH could potentially hinder proliferation of NSCs. Conversely, the downregulation of PTEN could facilitate the efficient activation of NSCs, potentially restoring neurogenesis. These findings underscore the critical role of maintaining PTEN homeostasis in preserving neurogenesis. It has also been reported that PTEN loss induced axonal regeneration thus promote neurological function restoration (Park et al., 2008; Hausott et al., 2022).

The alteration of niche after ICH led to many secondary injuries (Yan et al., 2022; Liu et al., 2023; Wu et al., 2023). Our data support that upregulation of PTEN after ICH inhibits NSC proliferation and impairs neurogenesis in the hippocampus. The result was confirmed by administration of PTEN inhibitor and conditional knock-out of PTEN. These expositions highlight the critical negative role of PTEN following ICH in maintaining neurogenesis.

In summary, our study for the first time provides direct evidence to show THF administration enhances cognitive recovery following ICH in mice. Mechanistically, THF suppresses the upregulation of PTEN expression, leading to elevated p-Akt and p-mTOR level, promoting the proliferation of NSCs, and subsequently ensures normal neurogenesis after ICH, ultimately alleviating cognitive impairment. These findings broaden our understanding of cognitive impairment after ICH and identify potential therapeutic strategies for post-ICH cognitive impairment.

## Synthesis

Reviewing Editor: Benjamin Deneen, Baylor College of Medicine Decisions are customarily a result of the Reviewing Editor and the peer reviewers coming together and discussing their recommendations until a consensus is reached. When revisions are invited, a fact-based synthesis statement explaining their decision and outlining what is needed to prepare a revision will be listed below. The following reviewer(s) agreed to reveal their identity: Michael Williamson.

The reviewers felt this paper is of potential interest, but is in need of a major revision that involves several key experiments and controls, as highlighted in great detail by Reviewer 1. In particular, testing of motor function after stroke is critical as it can impaired motor function can impact performance in cognitive tests. There were come concerns about the long lasting effects on neurogenesis and additional explanations are needed to validate this phenomenon. There are also concerns about the group size, as some of the behavioral studies need to have more power. Finally, some of the data presentation is confusing and needs to be clarified. As an example, the phrasing "PTEN knockout" was considered misleading. These and other key revision experiments are necessary for this manuscript to be considered any further.
